# Using the RE-AIM framework to evaluate physical activity public health programs in México

**DOI:** 10.1186/s12889-015-1474-2

**Published:** 2015-02-19

**Authors:** Edtna Jauregui, Ann M Pacheco, Erica G Soltero, Teresia M O’Connor, Cynthia M Castro, Paul A Estabrooks, Lorna H McNeill, Rebecca E Lee

**Affiliations:** Institute de Ciencias Aplicadas a la Actividad Física y Deporte, Centro Universitario de Ciencias de la Salud, Universidad de Guadalajara, Guadalajara, Mexico; Departamento Medicina Preventiva, Secretaria de Salud, Jalisco, México; Texas Obesity Research Center, Department of Health and Human Performance, University of Houston, Houston, TX USA; USDA/ARS Children’s Nutrition Research Center, Department of Pediatrics, Baylor College of Medicine, Houston, TX USA; Stanford Prevention Research Center, Stanford University School of Medicine, Stanford, CA USA; Virginia Polytechnic Institute and State University, 295 West, Blacksburg, VA USA; University of Texas MD Anderson Cancer Research Center, 1400 Pressler Street, Unit 1440, Houston, TX 77030 USA; College of Nursing and Health Innovation, Arizona State University, 550 N 3rd St, Phoenix, AZ 85004 USA

## Abstract

**Background:**

Physical activity (PA) public health programming has been widely used in Mexico; however, few studies have documented individual and organizational factors that might be used to evaluate their public health impact. The RE-AIM framework is an evaluation tool that examines individual and organizational factors of public health programs. The purpose of this study was to use the RE-AIM framework to determine the degree to which PA programs in Mexico reported individual and organizational factors and to investigate whether reporting differed by the program’s funding source.

**Methods:**

Public health programs promoting PA were systematically identified during 2008–2013 and had to have an active program website. Initial searches produced 23 possible programs with 12 meeting inclusion criteria. A coding sheet was developed to capture behavioral, outcome and RE-AIM indicators from program websites.

**Results:**

In addition to targeting PA, five (42%) programs also targeted dietary habits and the most commonly reported outcome was change in body composition (58%). Programs reported an average of 11.1 (±3.9) RE-AIM indicator items (out of 27 total). On average, 45% reported reach indicators, 34% reported efficacy/effectiveness indicators, 60% reported adoption indicators, 40% reported implementation indicators, and 35% reported maintenance indicators. The proportion of RE-AIM indicators reported did not differ significantly for programs that were government supported (*M* = 10, SD = 3.1) and programs that were partially or wholly privately or corporately supported (*M* = 12.0, SD = 4.4).

**Conclusion:**

While reach and adoption of these programs were most commonly reported, there is a need for stronger evaluation of behavioral and health outcomes before the public health impact of these programs can be established.

**Electronic supplementary material:**

The online version of this article (doi:10.1186/s12889-015-1474-2) contains supplementary material, which is available to authorized users.

## Background

Physical inactivity is endemic in Mexico and has become a public health priority. Findings from the National Health and Nutrition Survey (*Encuesta Nacional de Salud y Nutrición*, ENSANUT) reported that 19.4% of Mexican adults are physically inactive, with many more not meeting recommended guidelines. This represents a worrisome upward trajectory of an increase 44% from 2006 to 2012 [1,2]. High levels of physical inactivity have significantly contributed to the alarming rates of obesity and non-communicable diseases in Mexico [3]. Over 70% of the Mexican adult population and 26% of children are considered overweight or obese [2,4]. Furthermore, low levels of physical activity have led to increased rates of cardiovascular diseases and Type 2 diabetes, which are the two main causes of adult mortality in Mexico [5].

Public health campaigns that foster health and welfare in Mexico are important not only to improve health and welfare in Mexico, but also in the US, particularly among Mexican Americans, the largest group of Hispanics in the US. The Latino population in the U.S. has grown over 50% in the past decade with Mexicans comprising three quarters of this increase [6,7]. It is estimated that by the year 2060, one in three Americans will be of Latino or Hispanic origin; thus, programs that help to maintain the health of the Mexican public are very important to the health of the future American public [8].

Public health programs and initiatives are recognized as important, population-level strategies for promoting physical activity and improving health outcomes related to physical activity such as diabetes, heart health, cholesterol, and body composition in adults and children [9-12]. Public health programs have been widely used in Latin American countries, perhaps the most widely recognized is Ciclovia Recreativa, an open streets bicycling initiative [13]. Public health programs like Ciclovia capitalize on existing public spaces such as streets and parks, or existing community centers and physical activity resources [13,14]. These programs are capable of initiating positive changes at the individual, social, environmental, and policy levels, bringing together a diverse mix of key stakeholders such as community organizations, merchants, residents, and city officials [13-15]. Programs are often implemented by community health workers or *promotores*/*promotoras* and paid program employees [9,11,16,17]. Funding for these program resources is typically provided by government agencies or private industries. Many multinational beverage corporations have given a significant amount of funding to support physical activity programs and initiatives in Latin America, including Mexico. For example, Coca Cola is currently the largest funder of physical activity programs in Mexico [18].

Public health programs in Mexico and the U.S. have been well received and widely implemented; however, there have been few systematic investigations about the public health impact of these programs [4,19]. To produce a public health impact, physical activity programs need to measure their population reach while also assessing effectiveness across subgroups within the population [20,21]. Unfortunately, the evaluation of most public health physical activity programming is limited to measures of attendance and self-reported surveys measuring participant satisfaction, which provide valuable information, but are insufficient to determine public health impact [22].

Programs that can demonstrate a strong public health impact are often considered for broad dissemination to other communities, systems, and regions. However, programs with evaluation methods that do not include assessments of key areas can hinder successful dissemination, because there is no information about information on the expertise of those delivering the program, the program components, implementation activities and costs, the long-term sustainability of the programs and health and behavior outcomes for participants [10]. Community wide public health programs and initiatives are promising strategies for promoting physical activity among Mexicans; yet, the lack of evidence supporting these programs limits the ability to implement these programs on a broader state or national scale [9,13].

The RE-AIM framework is an evaluation approach that balances individual and organization-level factors that can then provide evidence about the public health impact of programs and information for other communities, organizations, or regions interested in replicating promising practices [22]. RE-AIM is an acronym that stands for reach and effectiveness at the individual level, adoption and implementation at the organizational level, and maintenance at the individual and organizational levels [23]. From a research perspective, the goal of RE-AIM is to provide a balanced assessment of internal and external validity factors. From a practice perspective, the goal of RE-AIM is to provide the information necessary for educators and organizations to make informed program adoption and implementation decisions based on the degree a program can reach the target audience, effectively change and sustain outcomes, be adopted and implemented in a wide variety of settings at a reasonable cost, and be sustained over time [24]. In this paper we take a practice perspective of the RE-AIM framework to better understand individual and organizational factors that can provide information on the public health impact and replicability of publically available physical activity programs and initiatives in Mexico. Due to the increasing Mexican and Mexican American population in the U.S., it is critical to establish culturally appropriate programs aimed at increasing levels of physical activity among this population that can achieve a public health impact and be scaled to other communities, organizations, or regions. A critical first step in establishing an evidence base is to determine whether necessary variables to begin to establish the evidence are available. The purpose of this study was to determine the degree to which PA programs in Mexico reported on individual and organizational factors using the RE-AIM framework and to investigate whether reporting differed by funding support for the program.

## Methods

### Identification of programs

We systematically identified publicly available programs that promoted physical activity between 2008 and 2013 in Mexico. The five year timeframe was chosen to capture programming before and after the 2011 national election which reflected a 2012 change in national leadership. Publicly available programs that had an accessible website and promoted physical activity in any population group were included. Programs had to be ongoing and actively engage in the community. Programs that promoted a single event (e.g., a specific footrace), national guidelines or agreements (e.g., Acuerdo Nacional de la Salud Alimentaria), websites with general health tips or advice, and results of national surveys or programs that did not include physical activity were not considered. Search terms associated with increasing physical activity were used in Spanish to identify programs in the Google search engine: programas (programs), activación física (to increase physical activity), México, niños (children), adultos (adults), escuelas (schools), ejercicio (exercise), trabajo (work). Journal articles from a companion review of physical activity interventions in Latin American populations that coincided with the development of this manuscript were also searched for possible Mexican programs that met inclusion criteria [4].

### Program coding

Program websites were examined to determine whether programs were administered at the national, state, community or individual level. Code sheets identified the population group focus (e.g., youth, women), any specific behavior change strategies that were used in the program (e.g., self-management, social support, self-efficacy), additional behaviors that might have also been promoted (e.g., dietary habits, screen time), behavioral target outcomes (e.g., physical activity increases) and changes in BMI.

A RE-AIM coding sheet was developed similar to others that have been used to review research-initiated behavioral interventions [4]. The questions that were included to provide a comprehensive evaluation of the reporting of RE-AIM indicators is presented below. Questions related to reporting of reach covered information on whom the program intended as participants, demographic and behavioral information for the target population, how the program was marketed (e.g. recruitment strategies that were used), and how well the program recruited the target population. Reporting of effectiveness of the programs was rated on the degree to which there was an evaluation that demonstrated changes in physical activity and the quality of the methods to assess physical activity. These included whether the program included an evaluation and any results including how many participants completed the program, what the program defined as a successful outcome, and any qualitative information that might have been used to evaluate the success of the program. Reporting of program adoption was explored using questions related to the location and delivery staff associated with the program, how the location was selected as well as determining the proportion of locations and agents that could have delivered the program that actually did. Reporting of implementation focused on the structure of the program, whether the program was delivered as intended, number and duration of sessions delivered, core program content, information on challenges and guiding theory. Reporting of maintenance was assessed at the organizational level using questions about the length of delivery and possible adaptions such as whether the program was still in operation or how it was sustained, reasons for discontinuation or modification of the program and information on implementation.

All information was entered into the code sheet. Information that was available was entered as yes if it were present, along with a brief description, or no, not reported on the website. A research assistant attempted to contact website hosts where information was not reported to confirm that it was not available. In cases where it was available, it was also recorded on code sheets. The University of Houston Committee for the Protection of Human Subjects reviewed and approved all procedures and determined that no consent was required for this study as no protected, participant data were collected.

Two native Spanish language speaking reviewers independently screened candidate websites to determine eligibility. Disagreements were discussed with the PI and four members of the research team and resolved by consensus. The original search produced 23 possible websites. Eleven websites did not meet inclusion criteria. Twelve programs met the inclusion criteria and were included in the final review.

Two reviewers coded each program for the presence or absence (Yes-present or No-absent) of the behavioral, outcome and RE-AIM indicators described above. Reviewers met to discuss any discrepancies in coding; resolution was completed by direct reference to the program website.

#### RE-AIM coding sheet questions

*Reach*

1. Is there information on the target population (i.e., whom they intended to have participate or benefit from the program)?
2. Is there demographic and behavioral information available for the target population?
3. Is there information on how the program was marketed or what recruitment strategies were used?
4. Is there information on the demographics and behaviors of the participants?
5. Is there information on how well the program recruited from the target population?
6. Are there sources of information available to assess the initiative’s reach?

*Efficacy/Effectiveness*

1. Did the program include an evaluation?
2. If yes, what were the results?
3. If an evaluation was complete, can you determine how many participants completed it?
4. Can you determine how the organization defined success of the program?
5. Did they use qualitative methods to determine effectiveness?

*Adoption*

1. Where did the initiative take place?
2. Describe the program location.
3. Describe who delivered the program (or implemented a policy).
4. Provide any information on why these locations were selected.
5. Could anyone deliver the intervention or were there restrictions? (please describe)
6. If you can determine the proportion of locations that had access to the program and actually delivered it, provide that number.
7. If you can determine the proportion of staff that had access to the program and actually delivered it, provide that number.

*Implementation*

1. If you have any information on how closely the program was delivered as intended add it here.
2. Provide any information that is available on the number of sessions, duration of each session, and frequency of sessions.
3. If available, describe the core program content.
4. Provide any qualitative information that is available on challenges or successes with the implementation process.
5. Was there a theoretical framework identified that was used to develop the program?

*Maintenance*

1. Is the program still in place? And if so, what information is available on why it was sustained?
  A. If no, reason for discontinuation
  B. If yes, was the program modified? Specify what was modified
2. Was the program institutionalized?

### Analyses

Descriptive information was tabulated across programs. Data exploration included frequency counts and percentages across the RE-AIM indicators. Programs were classified as completely government supported versus partially or completely privately or corporately supported. T-tests were calculated to determine whether the extent of RE-AIM indicators reported differed by government versus partial or complete private or corporate support. The data are presented in an additional file [see Additional file 1].

## Results

Of the 12 programs that were evaluated, seven (58%) were framed at the national level, with two (17%) of these having additional regional (state or local) programs that were affiliated with the national program. Table 1 presents the programs and their funding and support sources. The remaining programs were framed at the regional level, either state (*n* = 3, 25%), local (*n* = 1, 8%) or both (*n* = 1, 8%). Five (42%) were government supported, with two (17%) solely privately or corporate supported and five (42%) supported through a combination of government and corporate/private funding.

**Table 1:** **Programs and their funding and support organizations**

**Program Name**	**Funding and Support Organizations**
1. Vive Saludable Escuelas	PepsiCo Mexico; National Ministry of Education; National Commission of Physical Culture and Sports (CONADE); Latin American Institute for Educational Communication; Arturo Resenblueth Foundation; Union of Entrepreneurs for Educational Technology (UNETE)
2. Actívate México	Viviendo Positivamente; Coca Cola; National Commission of Physical Culture and Sports (CONADE); Confederacion Deportiva Mexicana; Instituto Slim de la Salud; Usada Health Sciences; EVAF; Specialized; Exercise is Medicine; Body System; Physical Activity Network of the Americas (RAFA/PANA); Agita Mundo; America College of Sports Medicine
3. Programa Estatal de Activación Física Estado de Nuevo Leon	Nuevo Leon State Institute of Physical Culture and Sport, Ministry of Health
4. Programa Muévete y Métete en Cintura	Federal Ministry of Health; Federal District (DF) Public Health Services
5. Agita Ags, Actívate	National Commission of Physical Culture and Sports (CONADE); Aguascalientes Ministry of Social Integration
6. Cinco pasos	Federal Ministry of Health; Federal Ministry of the Navy; Federal Ministry of Defense; Mexican Social Security Institute, National System of Integrated Family Change (DIF); PEMEX; Fundacion Franco-Mexicana para la Medicina IAP
7. Programa Escuela y Salud	Federal Ministry of Health Departments of Health and Public Education;
8. Programa Deporte Adaptado y Adulto Mejor Comude Zapopan	Zapopan Municipal Counsel of Sports
9. Juego y Comida dan salud a tu vida	Kelloggs; Ogali Consultoría en Nutricón; Project Concern International Mexico; MetLife Foundation.
10. Copa Coca Cola	Coca Cola; Fédération Internationale de Football Association (FIFA); Federación Mexicana de Fútbol Asociación (FEMEXFUT)
11. 11 Jugadas para la Salud	Fédération Internationale de Football Association (FIFA); Federal Ministry of Health; Federal Ministry of Public Education, Federación Mexicana de Fútbol Asociación (FEMEXFUT)
12. Zafo no Jugar	Coca Cola, Public Education Secretary; National Commission of Physical Culture and Sports (CONADE)

Targeted behaviors and measured outcomes of the programs are presented in Table 2. All (100%) programs included physical activity as a targeted behavior, as this was necessary for inclusion in the study; however, many programs also targeted additional behaviors. Five (42%) programs targeted dietary habits, and three (25%) specifically targeted increasing fruit and vegetable consumption. Two (17%) targeted reducing sugar sweetened beverage consumption, and one (8%) targeted reducing sedentary time. No programs targeted sleep.

**Table 2:** **Program target behaviors and outcomes assessed**

	**1**	**2**	**3**	**4**	**5**	**6**	**7**	**8**	**9**	**10**	**11**	**12**
Target Behaviors												
Physical Activity	x	x	x	x	x	x	x	x	x	x	x	x
Dietary Habits	x			x			x				x	x
Sleep												
Sedentary Activity										x		
Fruit Vegetable Intake						x			x			x
Sugar Sweetened Beverages							x					x
Behavioral and Physiological Outcomes Assessed												
Weight		x		x		x			x			
Height		x		x		x			x			
BMI				x		x						
Waist Circumference				x								
Smoking		x				x						
Alcohol use						x						
Physical Activity Engagement		x				x			x			
Other						x			x			

*Note*. 1 = Vive Saludable Escuelas; 2 = Actívate México; 3 = Programa Estatal de Activación Física Estado de Nuevo Leon; 4 = Programa Muévete y Métete en Cintura; 5 = Agita Ags, Actívate; 6 = Cinco pasos; 7 = Programa Escuela y Salud; 8 = Programa Deporte Adaptado y Adulto Mejor Comude Zapopan; 9 = Juego y Comida dan salud a tu vida; 10 = Copa Coca Cola; 11 = 11 Jugadas para la Salud; 12 = Zafo no Juga.

Four programs measured behavioral or physiological outcomes, the most common of which was change in body composition, with all four programs using weight (33%), two using BMI (17%), and one (8%) using waist circumference as outcomes that could be measured in the program. Only three (25%) had an outcome of increased physical activity engagement. Two (17%) also had outcomes focused on reducing smoking, and one (8%) on reducing alcohol use. One (8%) program had increasing water and fruit and vegetable consumption as an outcome corresponding with a program target behavior, and another (8%) had improving school environments as an outcome.

On average, program websites reported on 11.1 (±3.9) of the 27 RE-AIM indicator items, with a range of 3 to 17 indicators reported (Table 3). The proportion of reach indicators reported across programs was 45%, with an average of 2.7 (SD = 1.6), ranging from 0 to 5 out of 6 possible. Reach indicators included a description of the intended target population (*n* = 11, 92%), demographic and/or behavioral information about the target population (*n* = 6, 50%), marketing and recruitment strategies (*n* = 6, 50%), inclusion (*n* = 6, 50%), recruitment efficiency (*n* = 3, 25%) and other sources of information available (*n* = 5, 42%). The reporting of efficacy/effectiveness components was 34% across programs on average. Efficacy/effectiveness indicators included whether the program included an evaluation (*n* = 6, 50%), whether evaluation results were presented (*n* = 3, 25%), number of participants who completed the evaluation (n = 2, 16%), a program defined measure of success was presented (*n* = 3, 25%) or the use of qualitative methods (i.e. participant testimonials) (*n* = 3, 25%). The proportion of adoption indicators reported across programs was 60%. All (100%) websites identified where the program occurred, with most (*n* = 10, 83%) providing some description of the location. In contrast, few (*n* = 3, 25%) provided information on why locations were selected. Level of expertise of the staff that delivered the program was reported in 4 (33%) of the programs. Four (33%) programs reported the proportion of locations that had access to the program and delivered it, and one (8%) program reported the proportion of staff that had access to the program and actually delivered it. The average reporting proportion of implementation indicators across programs was 40%. No studies reported information on delivery as intended. Five websites (42%) reported information on dose (e.g., duration) of programs. All (100%) programs described core content, and four (33%) provided a rationale for the program (e.g., reduces chronic degenerative disease, overweight or obesity). No programs provided qualitative information on implementation. The average reporting proportion of maintenance indicators across programs was 35%; however, five programs did not report any maintenance indicators, primarily because they were no longer active. Only one (8%) program had been institutionalized.

**Table 3:** **Number of RE-AIM indicators (** ***n*** **= 27) reported by each program**

**Program**	**Year of inception**	**Reach (** ***n*** **= 6)**	**Effectiveness/Efficacy (** ***n*** **= 5)**	**Adoption (** ***n*** **= 7)**	**Implementation (** ***n*** **= 5)**	**Maintenance (** ***n*** **= 2)**	**Total (** ***n*** **= 27)**
Vive Saludable Escuelas	2003	4	1	5	2	1	13
Actívate México	2010	4	3	5	1	2	15
Programa Estatal de Activación Física Estado de Nuevo Leon	2010	0	1	3	2	0	6
Programa Muévete y Métete en Cintura	2008	2	0	5	2	0	9
Agita Ags, Actívate	2011	4	1	5	1	1	12
5 pasos	2010	2	2	4	2	1	11
Programa Escuela y Salud	2007	1	3	3	2	0	9
Programa Deporte Adaptado y Adulto Mejor Comude Zapopan	2011	2	1	7	3	1	14
Juego y Comida dan salud a tu vida	2006	3	3	4	3	0	13
Copa Coca Cola	1998	5	4	4	3	1	17
11 jugadas por la salud	2011	1	0	1	1	0	3
Zafo no jugar	2007	4	1	4	2	1	12
Mean (standard deviation)		2.7 (1.6)	1.7 (1.3)	4.2 (1.5)	2.0 (0.7)	0.7 (0.7)	11.1 (3.9)

T-tests suggested that the proportion of RE-AIM indicators that were reported for the programs did not differ significantly for those programs that were government supported (*M* = 10, SD = 3.1) from those that were partially or wholly privately or corporately supported (*M* = 12.0, SD = 4.4). As well, there were no differences by source of support for any of the individual indicators (all *p*s > .05).

## Discussion

This manuscript presents the evaluation of twelve public programs that promoted physical activity in Mexico, described the behavioral targets and outcomes of the programs, and determined the degree to which programs reported on individual and organizational factors using the RE-AIM framework. We also explored whether reporting differed by funding support for the program and found no difference in reporting between government versus privately supported programs. Perhaps the most striking finding was the lack of measurement of outcomes across programs. Although all programs had a behavioral target of promoting physical activity, only three had a measurement outcome of the program of increasing physical activity, suggesting that there was little acknowledgement of the relationship between promoting a behavior and measuring whether it was done. Four programs focused on body composition changes as outcomes, which may reflect the recent acknowledgement of the significant burden of overweight and obesity in Mexico.

Echoing the recognition of the burden of overweight and obesity, half of the programs also included behavioral targets focused on diet or nutrition, either dietary modification in general, or eating more fruits and vegetables or reducing sugar sweetened beverages in specific. One included reducing sedentary time, and none had any emphasis on sleep, despite the role of these factors in their relationship to overweight and obesity [25-27]. In addition to the body composition outcomes noted above, two programs had smoking reduction as an outcome, and one program also included outcomes focused on decreasing alcohol consumption and increasing water consumption. The lack of focus of programs on increasing water consumption may be related to the history of unsafe drinking water in Mexico [28,29]. This history endures even today, where it is socially questionable to invite people to drink water, and, instead, other beverages, often high in calories and low in nutrients, are offered [18,30].

The lack of measurement of outcomes was also echoed by the measurement of efficacy/effectiveness, with over half of the programs reporting none or one of the indicators in this domain. Half of the programs had no evaluation plan in place, and nine did not have a clear indicator or definition of how program success would be described. Perhaps the lack of efficacy/effectiveness measurement is driven by the inability of public programs to measure behavioral or health outcome changes among such large segments of the population. Although national surveys of population health in Mexico have been conducted since 1995, questions measuring physical activity, rather than merely sports participation, had not been measured until recently [31]. It may also be that measuring efficacy is simply perceived as too big a challenge by public health practitioners. In a survey of public health practitioners implementing the National Physical Activity Plan in the US, most reported that changes from the plan were difficult to implement and impact of changes was hard to observe [32]. In another investigation of implementation of policy and programs in the USA impact was inconsistently reported, only about half of the time [33].

In contrast to the lack of efficacy/effectiveness reporting, the reporting of program reach was more consistently available, echoing studies done in other countries [33]. Perhaps defining a target population, and showing that the population was reached is more important for marketing and reporting to program supporters. Large corporations that support programming need venues for promoting their name and products, so clear and careful definition of the population is very important. It is possible that programs that are funded via private sources may have a stronger need for evaluation to document effectiveness in order to justify corporate funds allocated to programs and promotion of their good corporate citizenship. Government agencies that rely on voter satisfaction to ensure political stability may also need to reach a carefully selected segment of the population, although it is impossible to document whether this is the case.

Factors related to adoption were the most commonly reported of all the RE-AIM indicators. Nearly all programs reported the location of initiatives and programming and who would deliver programming. Presumably these would be important for consumers of the programs. Nevertheless, few programs reported indicators related to the proportion of sites or program delivery agents who were offered the program and actually delivered the program. In some cases, this was not feasible, if the program were delivered online; however, in other cases, it simply wasn’t information that programs presumably collected.

Nearly all the programs described the core program components, including information on for whom the program was intended, where they could do the program, and what the program featured. Most other indicators of implementation were not included. Indicators of maintenance were largely absent, either because the program was primarily over, without reporting maintenance, or there was no maintenance plan in place. Only one program showed evidence of having been institutionalized, by having staff roles and responsibilities center on program goals.

Strengths of this study include a carefully constructed coding protocol and thorough search in a virtually unexplored area of research. Limitations include a small sample size of programs, limiting the investigations of relationships among variables. Coding was dependent on whether programs had websites available, which may have excluded programs without websites, and the content of the websites. When information was not available on the websites, research staff made attempts to contact program staff to determine whether information was available and not posted. Future research is needed to determine other channels through which public health programs might be disseminated, and to determine better measurement strategies in order to evaluate their effectiveness.

The information collected using the RE-AIM framework has important implications for future research, policy, and practice. This study identified indicators within the RE-AIM framework where reporting from public PA programs might be improved. This information must be used to help guide the development and design of future PA programs in order to be able to include reporting indicators so that it is possible to measure the impact of these programs and how they can be replicated in other settings. Perhaps the biggest area for improvement is in the reporting of indicators for measurement and evaluation of program outcomes. The lack of indicators available in these arenas may be due in part to the overwhelming number of participants in national level programs. In another study evaluating the implementation of programming in the US, program implementers often believed that adding RE-AIM indicators to evaluation plans required special training or expertise, suggesting that simple educational strategies about the importance and ease of measurement might improve reporting in the future [34]. In order to make measurement efforts more feasible, program officials should foster partnerships and collaboration with university researchers who can apply scientific understanding to developing suitable strategies for evaluating program outcomes or physicians in community clinics to develop strategies for overcoming measurement barriers. Most people (95%) in Mexico receive health care in public clinics which are often seen as “one-stop shops” where patients can see a physician, complete necessary lab work, and receive health information [35]. Clinics are under the jurisdiction of the office of the state Secretary of Health (*Secretaría de Salud*), which is also responsible for public health programs, making this an ideal partnership. Public clinics are accessible in the community and have the equipment and trained staffing to coordinate evaluation efforts in conjunction with public health programs.

The RE-AIM framework can be used as an evaluation tool, it can also be used at the program’s conception to help guide the planning of the program to ensure adoption, successful implementation, and evaluation. Using the RE-AIM framework to guide the design and evaluation of public health programs can lead to the development of policies and standards that can increase the execution and reporting of RE-AIM indicators. This may produce important information on the effectiveness and replicability of these programs, by improving smaller program details like the allocation of program funding and training of program staff.

## Conclusions

The Mexican government and private organizations have worked together and independently to fund physical activity programs in Mexico to address increasing levels of physical inactivity. While these programs have demonstrated adequate reach and adoption, poor monitoring and evaluation of outcomes has limited our knowledge of the effectiveness of these programs. There is a strong need to improve efforts of evaluating behavioral and health outcomes. Without this knowledge, we are unable to replicate these programs in other settings with sizeable Hispanic populations. Use of the RE-AIM framework to develop and evaluate future programs will increase our understanding of program factors that contribute to effective program with broad reach, successful implementation, and long-term outcomes.

## Additional file

Additional file 1:
**Data and Characteristics of Programs.**
